# Maternal Nutritional Programming: Sex-Specific Cardiovascular and Immune Outcomes Following Perinatal High-Fat Diet Exposure

**DOI:** 10.3390/nu17091464

**Published:** 2025-04-26

**Authors:** Yasir Alsiraj, Hong Huang, Robin Shoemaker, Brandon Schanbacher, Margaret Murphy, Peter Giannone, John A. Bauer

**Affiliations:** 1 Department of Pediatrics, College of Medicine, University of Kentucky, 138 Leader Ave, Lexington, KY 40536-0200, USA; 2 Department of Pharmacology and Nutritional Sciences, College of Medicine, University of Kentucky, Lexington, KY 40536-0200, USA; 3 Division of Neonatology, Department of Pediatrics, College of Medicine, University of Kentucky, 138 Leader Ave, Lexington, KY 40536-0200, USA

**Keywords:** maternal high-fat diet, perinatal programming, cardiovascular function, sex differences

## Abstract

**Background:** The long-term effects of a perinatal high-fat diet on the cardiovascular function of offspring are not well elucidated. We hypothesize that perinatal exposure to a high-fat diet alters adult cardiovascular and immune responses in a sex-specific manner. **Methods:** Male and female offspring were born to perinatal high-fat (pHFD) or control diet (pCD)-fed C57BL/6 mothers and weaned to a control diet. Cardiovascular function (baseline and response to an acute isoproterenol stress test) was quantified at 8 weeks of age, and acute blood inflammatory response to a single low dose of lipopolysaccharide at 9 weeks of age. **Results:** Male pHFD offspring had identical baseline cardiovascular function compared to pCD mice but a blunted response to isoproterenol (20–45% reductions in cardiac output, stroke volume, and left ventricular fractional shortening). In contrast, baseline cardiovascular parameters were reduced in female pHFD compared to pCD offspring, but there was no effect of perinatal diet on response to isoproterenol. Concentrations of TNF-α and IL-6 in plasma two hours after a low-dose LPS administration were highest in female pCD mice. **Conclusions:** Perinatal high-fat diet exposure resulted in sex-specific adaptations in cardiovascular function and immune response. Female offspring displayed baseline impairments, whereas male offspring showed latent vulnerability under stress. These differences may reflect underlying hormonal or epigenetic mechanisms that diverge by sex. Future studies should examine the roles of sex hormones and gene regulation pathways to better understand these dimorphic outcomes. These findings emphasize the importance of maternal diet in shaping offspring cardiometabolic risks and highlight potential avenues for nutritional interventions during pregnancy.

## 1. Introduction

Recent studies indicate a high prevalence of high blood pressure and other cardiovascular risk factors in youth and adolescents [1]. Known contributors include diet and physical activity, socioeconomic factors, and genetic predispositions [2], and emerging evidence indicates that the pre- and perinatal environment is a potential risk factor for childhood and adult cardiovascular disease [3].

This concept is based on the developmental origins of health and disease, which assert that adverse environmental exposures during the pivotal stages of development, particularly the prenatal phase, can engender long-lasting alterations in organ structure and function, increasing the risk of chronic diseases later in life [4]. Historical studies by Barker et al. demonstrated that wartime-related prenatal caloric restriction was linked with low birth weight and poor lifetime health [5,6], whereas more recent evidence links perinatal over-nutrition and maternal high-fat diets with fetal and offspring disease risks [3,7]. Maternal consumption of a high-fat diet during pregnancy exposes the developing fetus to an excessive influx of fats and nutrients, which can precipitate a cascade of alterations in development, potentially leading to lasting consequences [8,9]. Evidence from studies in mice has demonstrated that maternal high-fat diets induced cardiac hypertrophy and impaired systolic function in 12-week-old offspring [10], and that switching to a standard chow diet at weaning could attenuate the deleterious effects of a prenatal high-fat diet in adult offspring [11].

In humans, a maternal high-fat diet is associated with abnormal cardiac remodeling and reduced cardiac function in offspring, and a number of randomized controlled trials have demonstrated a protective effect of maternal intervention against adverse remodeling in offspring [12,13]. Evidence demonstrates that cardiac remodeling in early adulthood serves as a predictor for future cardiovascular events [14], but the effects of early cardiac impairments in the offspring of mothers with obesity on cardiovascular disease in childhood (or later in life) have not been clearly established [15].

The interplay between biological sex and prenatal exposure to a high-fat diet is also an intricate aspect of developmental biology with significant implications for future health and disease susceptibility. Male and female fetuses may have different responses to excess nutrients and the metabolic challenges posed by a high-fat diet that impact early cardiovascular development. These differences involve complex hormonal, genetic, and epigenetic mechanisms that can be sex-specific [16]. There are examples of sex-based differences in pediatric cardiovascular risks [17,18,19]. While there are some preclinical studies of sex differences in cardiovascular parameters in male versus female offspring exposed to a maternal high-fat diet [20], sex-specific knowledge of cardiac development after adverse perinatal exposure (and implications for future susceptibility to disease) is limited. Moreover, our research team previously demonstrated an enhanced acute inflammatory response to LPS in high-fat-diet-fed mice [21], and whether these effects extend to offspring exposed to a perinatal high-fat diet is not known. We hypothesized that perinatal high-fat diet exposure would alter cardiovascular and immune function in a sex-specific manner. To test this, we utilized a murine model to assess cardiovascular function (at baseline and in response to an acute stress test) and immune response to low-dose LPS in adult male and female offspring born from mothers fed a high-fat or control diet during breeding and suckling. 

## 2. Methods

### 2.1. Experimental Animals

All procedures were in accordance with the guidelines of the National Institutes of Health and approved by the Institutional Animal Care and Use Committee at Nationwide Children’s Hospital, and were performed on C57BL/6 mice purchased from Jackson laboratories. All animals were housed in a sterile cage rack 90 system with HEPA-filtered air circulation (approximately 50 air changes/hour, Allentown Caging Inc., Allentown, NJ 08501, USA). Male and female breeding-age mice (all virgins) were fed ad lib either a high-fat diet (HFD; 4.1 kcal/g and 20, 45, and 35 kcal% of protein, carbohydrates, and fat, respectively; Research Diets, Inc., New Brunswick, NJ, USA, Cat#D12336) or a matched control diet (CD; 3.6 kcal/gm and 20, 70, and 10 kcal% of protein, carbohydrates, and fat, respectively; Research Diets, Inc., New Brunswick, NJ, USA, Cat#D12337) throughout breeding. Pregnant females were then housed individually and maintained on the same diet throughout pregnancy (HFD or CD) and afterward until the offspring pups were weaned from suckling at 3 weeks of age. Pregnant females were randomly assigned to receive either the high-fat diet (HFD) or the control diet (CD). Following weaning, all offspring were then provided the control diet. A total of 6–8 pregnant females per group were used, producing 15–21 offspring per sex/diet group for final analyses. At 8 weeks of age, each mouse pup was studied in regard to baseline cardiovascular status and cardiovascular stress response using standardized methodologies (see below) and then returned to their cage. The investigators performing cardiovascular measurements and analysis were blinded to the perinatal diet group assignment to reduce assessment bias. At 9 weeks of age (1 week after cardiovascular assessment), each mouse was treated with an intraperitoneal dose of LPS (1 mg/kg) to investigate the sub-lethal inflammatory response. Serial blood samples were collected at 0, 2, and 24 h post-LPS, and immediately afterward animals were euthanized via pentobarbital (100 mg/kg ip), and organ tissues were collected for study [21]. All treatments and assessments were staggered to minimize batch effects. Throughout the study, mice were monitored weekly for general health and body weight. A 20% reduction in body weight was established as a humane endpoint, though no animals reached this threshold.

### 2.2. Cardiovascular Assessment: Echocardiography, Aortic Blood Flow Velocity, and Isoproterenol Stress Test

As previously described, echocardiographic images were recorded and analyzed by a Sonos 5500 echocardiogram using an ultrasonic probe (15 MHz, Agilent Technologies, Santa Clara, CA, USA) optimized and dedicated to rodent studies [22]. Briefly, light inhalant anesthesia (0.25–1.5% isoflurane in 95% O_2_/5% CO_2_) was used, and normothermia was maintained by a heating pad. Mice were placed in the parasternal, short-axis orientation using two-dimensional transverse imaging, and the M-mode was used to capture three loops (approximately five beat cycles per loop) of data for each animal. Left ventricular (LV) systolic and diastolic internal dimensions were determined according to published techniques [23] and used to calculate the fractional shortening (FS) using the following equation: FS = [(LVIDd − LVIDs)/LVIDd] × 100%, where LVID refers to the LV internal dimension at the diastole (d) and systole (s). A continuous wave Doppler flow probe in a short-axis, suprasternal position was used to record ascending aortic flow waveforms (at least five beats per loop) for the determination of velocity time intervals in each animal, and peak aortic flow velocity is reported as the velocity-time integral per second (cm/s) [24,25].

Following the capture of imaging data at baseline conditions, a single intraperitoneal dose of isoproterenol (ISO, 2.5 mg/kg ip in 0.1 mL saline) was used to elicit a standardized beta-adrenergic chronotropic response, thus serving as a pharmacologically induced acute cardiovascular stress test [26]. Imaging loops were captured from 10 to 20 min post ISO, and loops at the peak achieved heart rate during this interval were used as acute response results [27].

Following euthanasia, the aortic root (i.e., the outflow tract of the aorta) was isolated, and a cross-sectional area was measured using light microscopy with area-calibrated digital image analysis (Image-Pro Plus; Media Cybernetics, Silver Spring, MD, USA). Stroke volume (SV) was calculated by velocity-time integral × aortic root cross-sectional area, and cardiac output (CO) was calculated by SV × heart rate, as previously described [24,25].

### 2.3. Blood Assays

Blood was obtained by retroorbital collection at 2 h and cardiac puncture at 24 h sacrifice (EDTA 5 mM; aprotinin 500 KIU/mL). Plasma was obtained following centrifugation (5000 rpm, 10 min, 4 °C) and stored at −80 °C until assay. Serum cholesterol concentrations were obtained using an enzymatic assay kit (ThermoTrace Cholesterol reagent, Cat# TR13421; ThermoDMA, 845 avenue G East, Arlington, TX 76011, USA) triglycerides (ThermoTrace Triglycerides reagent, Cat# TR23221; ThermoDMA, 845 avenue G East, Arlington, TX 76011, USA) cholesterol, LDL (EZ LDL™ kit, Cat# 358A, Sigma Diagnostics, INC. St. Louis, MO 63178, USA), and cholesterol, HDL (EZ HDL™ kit, Cat# 354L-A, Sigma Diagnostics, INC. St. Louis, MO 63178, USA).

### 2.4. Inflammatory Marker Measurements

Cytokine levels in blood samples and tissues were measured using enzyme-linked immunosorbent assays (ELISAs). ELISA kits specific for mouse TNF-α (BD BioSciences, Franklin Lakes, NJ, USA; CAT# 555268), IL-6 (BD BioSciences, Franklin Lakes, NJ, USA; CAT# 555240), and SAA (Biosource, International, Inc. Camarillo, CA, USA; CAT#KMA0012) were used according to the manufacturer’s instructions.

### 2.5. Histological Assessment of the Heart

After euthanasia, hearts were excised, rinsed in cold saline, and weighed. The left ventricle was then cross-sectioned at the mid-papillary level, fixed in 10% neutral-buffered formalin, and embedded in paraffin. Five-micrometer sections were prepared and stained with hematoxylin and eosin (H&E). Images were used to measure the left ventricular (LV) lumen area, the total LV cross-sectional area, and LV wall thickness. All measurements were performed by two independent, blinded investigators.

### 2.6. Statistical Analysis

All data are presented as mean ± standard error (SEM), where *n* = 15–21 per group. Sample size was based on experience from previous studies. Data were tested for normality using the Shapiro–Wilk test; if normality was violated, transformations and/or nonparametric tests were considered. To define differences between groups, we used two-way ANOVA tests, testing for both perinatal diet exposure effects and sex-based effects on each variable. An unpaired Student’s *t*-test was used for two-sample comparisons. Statistical significance was defined as *p* < 0.05. Statistical analyses were performed using GraphPad Prism 8.

## 3. Results

### 3.1. Body Weight, Organ Weight, and Blood Lipid Levels

**Figure 1:**
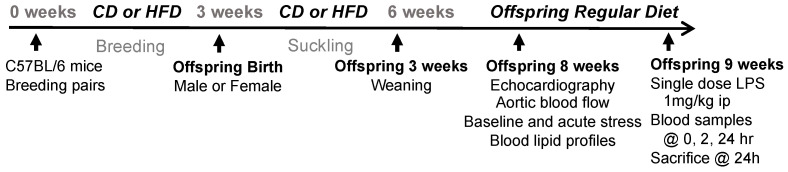
Study schematic. C57BL/6 breeding pairs were fed a high-fat or control diet, which was maintained through lactation. Male and female offspring were weaned onto the control diet for the remainder of the study. Blood lipid and cardiovascular assessments were performed at 8 weeks of postnatal age, and LPS was administered at 9 weeks of postnatal age.

Shown in Table 1 are body weights, organ weights, and blood lipid levels in male and female offspring at 8 weeks of age following perinatal high-fat diet (pHFD) or perinatal control diet (pCD) exposure (see Figure 1 for study schematic). Body weights were higher in males versus females (*p* < 0.05), but in males there was a slight reduction in body weight in the pHFD compared to pCD mice (*p* < 0.05). Organ weights were not different between perinatal diet groups or sex, with the exception of increased heart size (as a percentage of body weight) in females (*p* < 0.05). Total cholesterol and HDL levels were reduced in females compared to males (*p* < 0.05), but there were no effects of perinatal diet on total cholesterol, triglycerides, LDL, or HDL in either sex. These data indicate that perinatal HFD modestly reduced body weight in both sexes without significantly altering relative organ weights, except for minor sex-specific differences in heart and lipid profiles.

### 3.2. Baseline and ISO-Mediated Parameters of Cardiovascular Function

**Figure 2:**
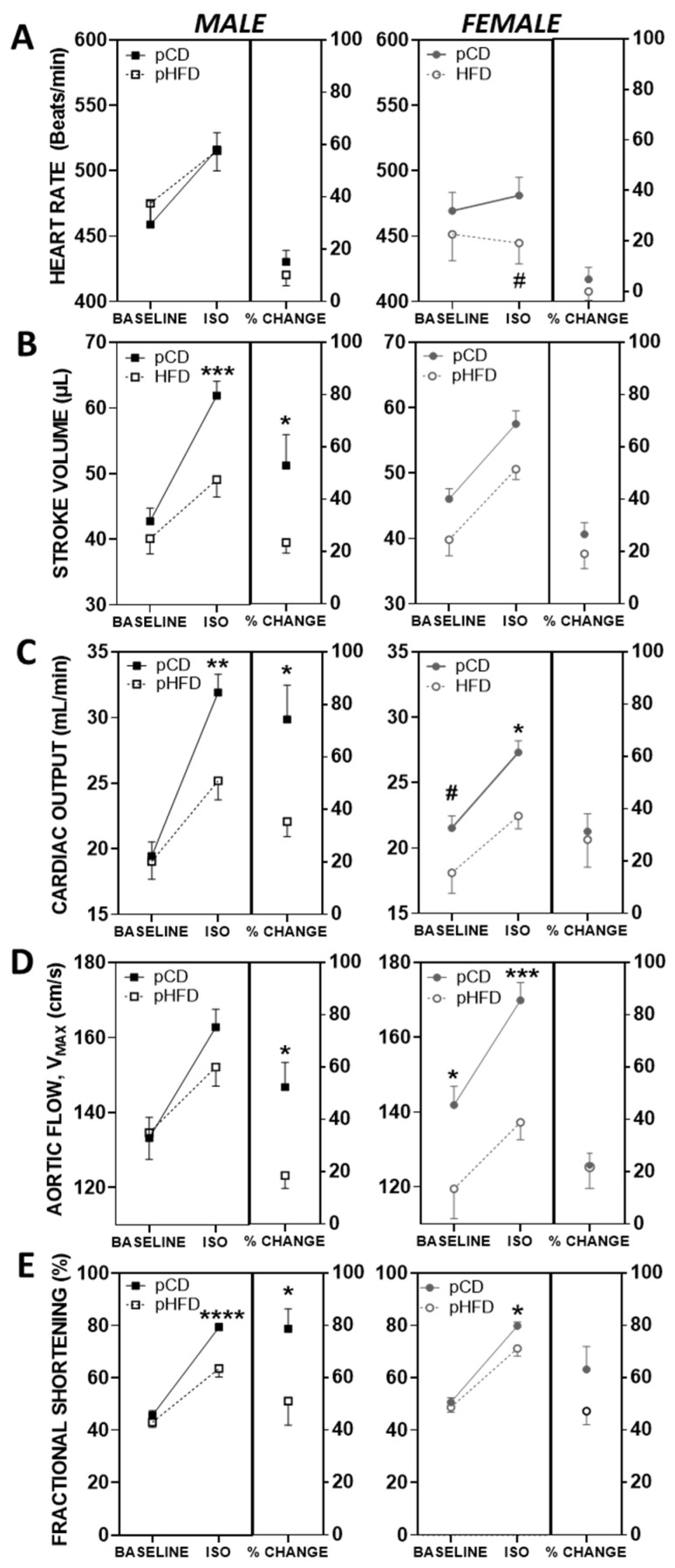
Baseline and ISO-mediated parameters of cardiovascular function in male and female mice exposed to a perinatal control diet (pCD) or perinatal high-fat diet (pHFD). (**A**) Heart rate, (**B**) stroke volume, (**C**) cardiac output, (**D**) peak aortic blood flow velocity, and (**E**) fractional shortening obtained by echocardiography and aortic Doppler waveform in 8-week-old male (**left panel**) and female (**right panel**) offspring that were prenatally exposed to either a control diet or a high-fat diet. For each parameter, data are expressed as mean ± SEM at each timepoint (baseline and after acute stress with isoproterenol) and percent change with isoproterenol. Male pCD, *n* = 21; male pHFD, *n* = 15; female pCD, *n* = 23; female pHFD, *n* = 17. *, *p* < 0.05; **, *p* < 0.01; ***, *p* < 0.001; ****, *p* < 0.0001 compared to pCD within sex and #, *p* < 0.05 compared to males within treatment by two-way ANOVA with multiple comparisons.

Figure 2 depicts the results of cardiovascular assessment (baseline, followed by acute ISO stress test) of male (left panel) and female (right panel) offspring mice at 8 weeks of age. In males, baseline cardiovascular parameters were not altered by perinatal diet. Administration of ISO to male mice resulted in a marked increase in heart rate, stroke volume, cardiac output, peak aortic blood flow velocity, and fractional shortening. However, the effect was blunted with perinatal HFD, where stroke volume, cardiac output, and fractional shortening were significantly reduced in male pHFD compared to pCD mice administered ISO (Figure 2B,C,E—left panel; *p* < 0.01). The attenuated ISO response in male pHFD offspring indicates a stress-dependent cardiovascular deficit.

In contrast to male mice, where there was no effect of perinatal diet on baseline cardiovascular parameters, female pHFD mice had significantly lower values for stroke volume, cardiac output, and peak aortic blood flow velocity at baseline compared to pCD (Figure 2B–D—right panel; *p* < 0.05). Unlike male mice, female pHFD mice did not exhibit differences in ISO-mediated parameters compared to pCD mice, despite having reduced baseline cardiovascular values (Figure 2B–E; *p* < 0.05). Heart rate did not increase with ISO in female pHFD mice and was lower than male pHFD mice (Figure 2A; *p* < 0.05). Despite reductions in absolute values of cardiac parameters in female pHFD compared to pCD mice, responsiveness to ISO was not different among groups. Unlike males, female pHFD offspring exhibit evidence of baseline cardiac dysfunction, but their functional reserve under adrenergic stress is preserved. This is in contrast to males, where the response to ISO in pHFD was significantly attenuated compared to the response in pCD mice for stroke volume, cardiac output, peak aortic blood flow velocity, and fractional shortening (*p* < 0.05 for each parameter assessed using a t-test for percent change with ISO between pCD and pHFD). Together, the results show that perinatal HFD disrupts cardiac function in a sex-specific manner, blunting stress responses in males and impairing baseline parameters in females.

### 3.3. Blood Immune Response to Acute LPS

**Figure 3:**
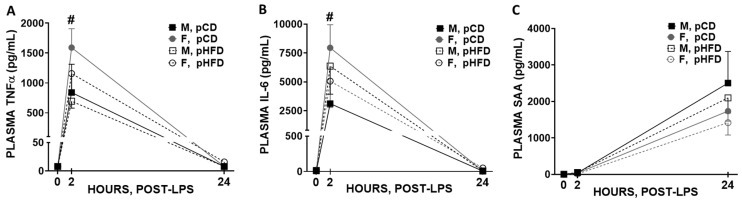
Plasma inflammatory responses to LPS in male (M) and female (F) offspring. A single dose of LPS (1 mg/kg body weight) was administered to 9-week-old male and female mice exposed to the high-fat or control diet during the perinatal period, and the following were quantified in plasma at 0, 2, and 24 h post-LPS: (**A**) TNF-α, (**B**) IL-6, and (**C**) SAA. Male pCD, *n* = 7; male pHFD, *n* = 8; female pCD, *n* = 14; and female pHFD, *n* = 9. Data are mean ± SEM. #, *p* < 0.05 for females compared to males within the pCD group by two-way ANOVA with multiple comparisons.

Following cardiovascular assessment, male and female pCD and pHFD offspring (9 weeks of age) were administered a single dose of LPS (1 mg/kg body weight). Shown in Figure 3 is the blood cytokine response (TNF-α, IL-6) and acute phase reactant SAA at timepoints of 0, 2, and 24 h after LPS treatment. In all groups, LPS resulted in significant elevations in the concentrations of TNF-α and IL-6 after 2 h, followed by a decline to pre-LPS levels after 24 h. Notably, female pCD mice had significantly higher concentrations of TNF-α and IL-6 at the 2 h time point compared to male pCD (Figure 3A,B; *p* < 0.05). SAA was significantly elevated in all groups at the 24 h timepoint (*p* < 0.0001), with a trend toward higher levels in male mice, although this did not reach statistical significance (Figure 3C). No statistically significant differences between perinatal diet exposure groups were observed in concentrations of any of the measured inflammatory markers. A sex-specific immune response to LPS was observed, with female pCD offspring mounting the strongest early cytokine surge. While not directly influenced by perinatal diet, the pattern suggests possible sex-dependent immune programming that may interact with maternal nutritional exposures.

### 3.4. Histological Assessment of Offspring Hearts

**Figure 4:**
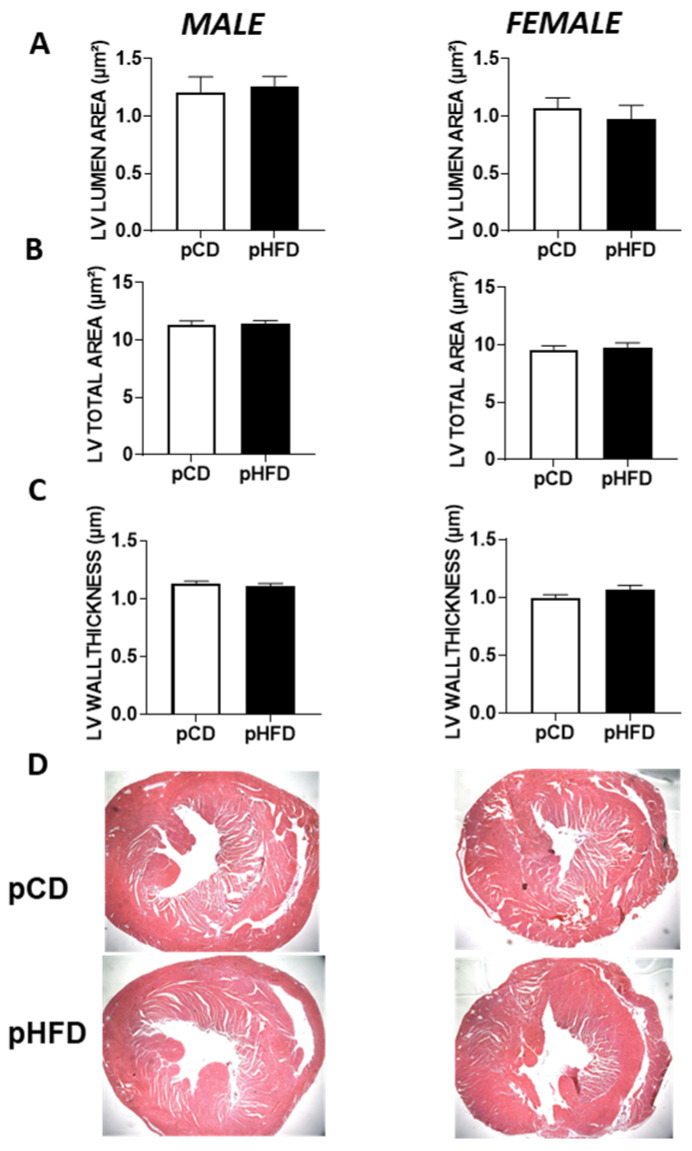
Histological characteristics of hearts in male vs. female offspring that were prenatally exposed to either the control diet or high-fat diet. (**A**) LV lumen area; (**B**) LV total area; (**C**) LV wall thickness; and (**D**) representative images of heart sections. Male pCD, *n* = 16; male pHFD, *n* = 12; female pCD, *n* = 19; and female pHFD, *n* = 9. Data are mean ± SEM.

To determine whether a maternal high-fat diet results in gross structural or morphological changes in offspring hearts by 8–9 weeks of age, we performed histological analyses (Figure 4). No significant differences were observed among pCD and pHFD groups in the LV lumen area (Figure 4A), the total LV area (Figure 4B), or the LV wall thickness (Figure 4C), regardless of sex. Representative H&E-stained images of each group are shown in Figure 4D. These findings indicate that, at least by 8 weeks of age, exposure to a maternal high-fat diet did not induce overt macroscopic or histological remodeling of the LV. This contrasts with the functional impairment seen in the pHFD male group under isoproterenol stress, suggesting that early functional alterations may occur without significant anatomical change.

### 3.5. Summary of Key Findings by Sex

Overall, these results support a sex-specific impact of perinatal high-fat diet exposure on offspring physiology. In males, baseline cardiovascular function was unaffected, but a blunted response to acute isoproterenol stress emerged, indicating latent functional impairment. In females, by contrast, baseline cardiac function was reduced with perinatal HFD, yet the degree of ISO responsiveness was not altered, suggesting early onset dysfunction rather than stress-induced vulnerability. Immune responses to LPS were also sex-specific, with female pCD mice displaying the highest pro-inflammatory cytokine levels at 2 h. No significant histological changes were detected in either sex, reinforcing the idea that functional abnormalities may precede structural remodeling in this model. Together, these data highlight a nutritionally programmed, sex-specific divergence in cardiovascular and immune trajectories following maternal high-fat diet exposure.

## 4. Discussion

### 4.1. Overview and Major Findings

Maternal obesity and high-fat diets are linked with adverse effects on cardiac developmental outcomes in offspring, but their long-term impact on cardiovascular risk remains poorly understood [28]. While several studies highlight this risk, few have investigated the sex-specific cardiovascular consequences of a maternal high-fat diet. Our study sought to fill this gap by investigating the cardiac functional parameters in male and female adult offspring with perinatal exposure to a high-fat or control diet at baseline and in response to an isoproterenol-induced acute cardiovascular stress test. Key outcomes include the following: (1) male pHFD offspring had similar baseline cardiovascular function but a reduced ISO-mediated response (stroke volume, cardiac output, and fractional shortening); (2) female pHFD mice showed reduced baseline stroke volume and cardiac output but no difference in ISO response magnitude versus female pCD mice; (3) plasma TNF-α and IL-6 levels at 2 h post-LPS were highest in female pCD mice; and (4) histology revealed no overt LV structural changes at 8–9 weeks, implying functional deficits, especially in pHFD males, may precede anatomical remodeling.

### 4.2. Cardiac Function and Sex-Specific Programming

The current study utilizes a mouse model in which breeding pairs and lactating dams were fed a high-fat or control diet, and offspring were weaned (and remained) on the control diet. In a related model of adverse metabolic imprinting, adult rat offspring exposed to early postnatal overfeeding exhibited increased body weight, blood pressure, ventricular hypertrophy, and impaired ex vivo cardiac function compared to controls [29]; another study reported impaired cardiac remodeling in obese adult offspring under similar conditions [30]. Another mouse study examined cardiovascular function in adult offspring born to mothers fed a high-fat diet before pregnancy and through lactation and subsequently weaned onto a chow diet (similar to the model used in the current study). At 12 weeks of age, Langendorf-perfused hearts from (non-obese) offspring with prenatal high-fat diet exposure exhibited reduced systolic and diastolic function [10]. Other groups have also reported sex differences in cardiovascular outcomes following maternal high-fat diet or overfeeding. For example, Vaughan et al. observed diastolic dysfunction in the offspring of HFD-fed dams, which progressed in females but not males over two years [20]. While their study found no differences in systolic function at 3 months, functional decline emerged later, highlighting the importance of long-term follow-up. These findings are consistent with our observed baseline impairment in female pHFD offspring and emphasize a potential window of latent vulnerability that may evolve differently by sex over time.

In contrast, other studies have reported cardiac dysfunction in male offspring after maternal high-fat diet exposure. A recent study in a rat model of maternal high-fat diet, where newly weaned offspring had cardiac mitochondrial abnormalities, reported impaired diastolic dysfunction in males only compared to age-matched offspring from lean rats [31]. Likewise, male rats, but not female, exposed to early overfeeding had increased blood pressure and ventricular wall thickness [32], and another study of rat offspring (45 days postnatal) after early overfeeding reported cardiac hypertrophy in both sexes; functional impairments were only evident in males [33]. In our study, 8-week-old male and female offspring exposed to a maternal high-fat diet exhibited impaired cardiac function. This is consistent with recent findings by Wang et al. (2022), who demonstrated that maternal HFD exposure increased the heart weight-to-body weight ratio and upregulated cardiac hypertrophy-associated genes, particularly in male offspring [34]. These findings are consistent with prior reports, though previous studies were primarily conducted in males. Our data extend previous findings by including both sexes, showing reduced baseline cardiac function in female pHFD mice, while male pHFD mice displayed a blunted cardiac stress response. This suggests sex-specific mechanisms of developmental cardiac programming. Data from our studies can be taken into context with studies of sex differences in humans, where males are more likely to develop heart failure with systolic dysfunction and reduced ejection fraction, and females are more likely to develop heart failure with diastolic dysfunction and preserved ejection fraction that is augmented by obesity [35,36].

### 4.3. Histological Findings and Structural Insights

In line with the observed functional impairments, we also conducted a histological assessment of the left ventricle (Figure 4). Interestingly, no significant morphological changes were apparent at 8–9 weeks in the pHFD vs. pCD offspring. These data suggest that the reduced functional response to isoproterenol observed in the male pHFD group may reflect more subtle alterations in cardiac cellular or molecular mechanisms rather than gross anatomical remodeling. Indeed, it is possible that functional deficits manifest earlier than overt structural changes or that progressive alterations might arise later in adulthood. Our results thus highlight a scenario in which a maternal high-fat diet can lead to latent cardiac vulnerability unmasked by acute stress testing, even when standard histology shows no overt pathology. Future studies might incorporate additional microscopic or molecular assays like fibrosis staining, collagen quantification, or ultrastructural electron microscopy to reveal microstructural changes not detectable by H&E alone. Recent studies have demonstrated that a maternal high-fat diet can induce significant transcriptomic and metabolic shifts in the developing heart. For instance, Nirala et al. (2024) reported upregulation of key lipid metabolism genes in embryonic and neonatal mouse hearts [37], while Preston et al. (2020) found that maternal HFD and diabetes upregulated mitochondrial biogenesis pathways in newborn rat hearts [38]. Together, these findings highlight that molecular and epigenetic alterations may precede detectable anatomical remodeling, supporting the idea of early, sex-specific cardiac vulnerability. We focused on the adolescent stage (~8–9 weeks), as recommended in prior murine cardiometabolic work. However, a longitudinal approach tracking older offspring (e.g., 6–12 months) might reveal whether these sex-specific patterns persist or worsen.

### 4.4. Immune Outcomes and Inflammatory Response

Previous studies from our lab demonstrated a heightened immune response to low-dose LPS in female mice fed a high-fat versus regular chow diet [21], and we sought here to determine whether this effect extends to offspring with maternal high-fat diet exposure. Clinically, cardiac dysfunction drives sepsis mortality, and sepsis mortality rates tend to be higher in males [39], but mechanisms underlying differences in males and females are not clear. A recent study investigated sex differences in resilience to sepsis-induced cardiac metabolic dysfunction in mice and found that an LPS challenge of 10 mg/kg resulted in deterioration in cardiac electrical activity and function, along with severe cardiac injury, inflammation, and higher mortality in male mice compared to females [40]. Further, in adult rat offspring investigated for effects of pre-eclamptic fetal programming, sex differences in inflammatory and cardiovascular complications to endotoxemia were found, where male offspring specifically exhibited myocardial dysfunction [41]. In our study, administration of a low dose of LPS led to the highest levels of blood inflammatory markers in female adult offspring exposed to the control prenatal diet, which was significantly higher than males. While these data are in contrast to our previous study, where high-fat feeding led to a heightened inflammatory response in females, they do agree with published data that male sex hormones decrease cellular immune response, and estrogen may confer a protective response under septic conditions [42]. Our data suggest that maternal high-fat diet exposure in female mice may blunt the immune response to LPS, similar to the response observed in male mice of both diet exposure groups. Although the C57BL/6 mouse strain is widely used and offers experimental reproducibility, it may not fully reflect the complexity of human cardiometabolic disease, limiting translational applicability. Additionally, the study assessed cardiovascular and immune outcomes at a single adolescent timepoint; therefore, long-term consequences of perinatal high-fat diet exposure, including effects in adulthood or aging, remain unknown.

### 4.5. Translational and Public Health Implications

This study aligns with the concept of nutritional programming; maternal diet can shape offspring cardiometabolic risk across the lifespan. In addition to cardiovascular outcomes, altered lipid metabolism, disrupted nutrient transport, and epigenetic reprogramming during fetal development may contribute to metabolic and immune dysfunction later in life. The public health implications are significant: nutritional interventions during pregnancy, such as moderating fat intake and optimizing micronutrients, may reduce offspring susceptibility to cardiovascular and immune dysfunction. These findings support the integration of maternal dietary interventions into clinical care models, emphasizing nutritional counseling during pregnancy as a key preventive measure. These insights may inform dietary guidelines that offer non-pharmacologic strategies to mitigate early-life chronic disease risk. Although rodent models do not fully replicate human complexity, the observed sex-specific responses align with known patterns in human cardiovascular and immune health. These findings emphasize the translational relevance of maternal dietary interventions during pregnancy and lactation. Integrating results from this model into prenatal care guidelines could be especially beneficial for individuals with obesity or metabolic syndrome. We focused on the adolescent window (8–9 weeks), a well-accepted stage for detecting early cardiometabolic phenotypes. However, long-term follow-up studies are needed to evaluate whether these impairments persist or progress over time. The findings suggest that male offspring may exhibit a unique pattern of vulnerability but also potentially greater responsiveness to interventions designed to counteract the effects of a high-fat prenatal environment.

### 4.6. Mechanistic Drivers of Sex-Specific Programming

Differences in sex hormones, genetics, and epigenetics may underlie the observed sex differences in our study [43]. Sex hormones such as estrogen and testosterone are known to play significant roles in modulating cardiovascular health and may differentially influence the development of the cardiovascular system in males and females from prenatal stages onward [44]. For instance, estrogen is implicated in cardioprotective effects that could explain the robust cardiovascular response to stress observed in female offspring. Additionally, genetic differences between the sexes, particularly those associated with the presence of XX (in females) or XY (in males) sex chromosome complements, could contribute to divergent developmental courses in response to environmental factors like a high-fat prenatal diet. These chromosomal differences not only affect gene expression directly involved in metabolic and cardiovascular systems but may also influence epigenetic modifications [45]. Epigenetic mechanisms, including DNA methylation and histone modifications, are responsive to environmental inputs like nutrition and can lead to long-lasting changes in gene expression that differ between sexes [43]. Recent work by Li et al. (2024) further demonstrates that a maternal high-fat diet can promote cardiovascular disease in offspring through persistent epigenetic memory, linking maternal nutrition directly to programmed vascular risk [46]. These biological factors, including hormonal, genetic, and epigenetic factors, likely interact in complex ways to mediate the distinct cardiovascular responses observed in male and female offspring. While direct measurement of sex hormone levels (e.g., estrogen and testosterone) was beyond the scope of this study, we acknowledge their likely role and have identified them as key targets for future mechanistic investigations. In future work, we plan to investigate whether gene expression profiles or methylation patterns differ in male and female offspring, providing further insight into the sex-specific mechanisms by which a maternal high-fat diet affects cardiac function. Better understanding of the underlying mechanisms could enhance our ability to develop personalized medical strategies that effectively address the impacts of prenatal nutrition on lifelong health outcomes.

## 5. Conclusions and Future Directions

Male and female offspring respond differently to perinatal HFD exposure, with males showing stress-sensitive functional impairment and females showing baseline dysfunction. These sex-specific adaptations suggest complex, interacting hormonal, genetic, and epigenetic mechanisms. Future studies incorporating transcriptomics, hormone profiling, and longitudinal designs will deepen mechanistic insights and inform targeted prenatal nutritional interventions.

## 6. Limitations

Several limitations warrant a mention. First, we assessed offspring only at 8–9 weeks of age, which may have missed structural or functional changes that emerge earlier or later in life. Second, although our histological analysis did not reveal overt morphological differences using H&E staining, more subtle alterations, such as fibrosis, ultrastructural abnormalities, or cellular remodeling, may have gone undetected. Third, while we evaluated inflammatory responses to LPS at multiple time points (0, 2 h, and 24 h), the limited sampling intervals may not fully capture the kinetics of cytokine production or resolution phases. Lastly, we did not measure parameters such as circulating sex hormone levels, oxidative stress markers, or gene expression profiles. Future studies that include longitudinal follow-up, additional immune time points, and molecular analyses (e.g., transcriptomics, methylation, and signaling pathways) will help clarify how maternal high-fat diet programs sex-specific cardiovascular and immune outcomes.

## Figures and Tables

**Table 1:** Characteristics of offspring that were prenatally exposed to either the control diet or high-fat diet.

	Male Offspring		Female Offspring	
	Prenatal (Maternal) Diet			
Parameters	pCD	pHFD	pCD	pHFD
Body Weight (g)	21.68 ± 0.76	19.5 ± 0.5 *	16.0 ± 0.4 #	15.2 ± 0.4 #
Heart (%)	0.6 ± 0.01	0.62 ± 0.01	0.71 ± 0.019 #	0.69 ± 0.013 #
Liver (%)	5.04 ± 0.1	5.35 ± 0.1	5.1 ± 0.11	5.3 ± 0.12
Spleen (%)	0.44 ± 0.02	0.49 ± 0.02	0.44 ± 0.02	0.52 ± 0.019
Pancreas (%)	0.88 ± 0.03	0.82 ± 0.04	0.88 ± 0.033	0.90 ± 0.28
Total Cholesterol	166 ± 7.2	148 ± 4.9	110 ± 4.4 #	105 ± 2.5 #
Triglycerides	74 ± 7.8	69 ± 7.7	54 ± 3.1	59 ± 6.3
HDL	65 ± 1.8	65 ± 4.4	45 ± 1.7 #	50 ± 3.9 #
LDL	93 ± 4.8	86 ± 3.9	72 ± 3.6 #	72 ± 2.5

#, *p* < 0.05 compared to the corresponding sex; *, *p* < 0.05 compared to control diet using two-way ANOVA with multiple comparisons. Heart (%), liver (%), spleen (%), and pancreas (%) represent the ratio of the organ weight to the total body weight × 100.

## Data Availability

Materials and data described in the manuscript will be made available to researchers upon reasonable request by emailing the corresponding authors.

## Abbreviations

**Abbreviation**	**Full Term**
HFD	High-Fat Diet
CD	Control Diet
CV	Cardiovascular
pHFD	Perinatal High-Fat Diet
pCD	Perinatal Control Diet
ISO	Isoproterenol
LPS	Lipopolysaccharide
TNF-α	Tumor Necrosis Factor-alpha
IL-6	Interleukin-6
SAA	Serum Amyloid A
CO	Cardiac Output
SV	Stroke Volume
FS	Fractional Shortening
LV	Left Ventricle
M	Male
F	Female
ELISA	Enzyme-Linked Immunosorbent Assay
SEM	Standard Error of the Mean
ANOVA	Analysis of Variance
